# Issues of Recruitment and Rationale for Conducting Clinical Trials on Mutans Streptococci Suppression in Mothers

**DOI:** 10.1155/2010/107147

**Published:** 2010-08-10

**Authors:** Walter A. Bretz, Odila P. S. Rosa, Salete M. B. Silva, Patricia Corby, Lisa Weissfeld, Walter J. Loesche

**Affiliations:** ^1^College of Dentistry, New York University, NY 10010, USA; ^2^Faculdade de Odontologia de Bauru, Universidade de São Paulo, 17012-901 Bauru, Brazil; ^3^Graduate School of Public Health, University of Pittsburgh, Pittsburgh, PA 15261, USA; ^4^School of Dentistry, University of Michigan, Ann Arbor, MI 48109, USA

## Abstract

The aims of this study are (1) to describe issues related to recruitment of mothers participating in a clinical trial of transmission of mutans streptococci (MS) from mother to child in Bauru, Brazil and (2) to perform cross-cultural and temporal comparisons of levels of infection of the MS in mothers of Bauru. A total of 1422 mothers were visited at their domiciles. Cutoff levels for the MS were established at ≥10^5^ CFU/mL saliva. The main reason for a mother not enrolling was not being highly infected by the MS, yet 76% of mothers presented with levels ≥10^5^ CFU/mL saliva. Recent studies in industrialized countries showed a negative coefficient for linear tests indicating significant decline overtime in the levels of MS in mothers. Intercountry comparisons for mothers' salivary levels of the MS with the Bauru study as the reference revealed significant differences with studies conducted in the last two decades.

## 1. Introduction

Thirty years ago, Kohler and colleagues demonstrated that the suppression of high levels of the mutans streptococci (≥10^6^ colony-forming units (CFU)/mL of saliva) by means of comprehensive and tailored regimens delivered to mothers of newly born babies was attainable [1]. This suppression of high levels of the mutans streptococci (MS) in mothers resulted in a lower prevalence of the MS and of dental decay in their children as far as when these children were 7 years of age, when compared to children of mothers receiving symptomatic dental treatment [2, 3]. These high levels of infection in mothers in Sweden at that time (1978) would likely not be found in most communities of industrialized countries nowadays, probably due to the widespread use of antibiotics, the use of fluoridated dentifrices, the introduction of dietary modifiers such as sugar substitutes, and public health efforts [4].

A number of investigators from industrialized countries have reported the levels of the MS in mothers and/or pregnant mothers throughout time [5–8]. Nonindustrialized countries have not fully experienced the benefits of measures that might interfere with the levels of the MS compatible with the diagnosis of an infection. In Brazil, high levels of the MS and of dental caries are still found in children and adults [9–15]. 

An inadequate sample can significantly alter the endpoints of clinical trials. The studies conducted by Kohler and colleagues suffered from methodological issues related to the design and implementation of clinical trials that include lack of randomization and of placebo-based groups which could have potentially biased study results. We have designed and implemented a randomized double-blind placebo-based clinical trial to look at the effects of combined chemical modalities on the suppression of MS in mothers and the consequences of these efforts on dental caries onset in their children. To date, there have been no reports describing issues of recruitment and of comprehensive data reporting details about potential groups of mothers to be enrolled in studies of the suppression of MS infections. 

The specific aims of this paper are twofold: (1) to describe issues related to recruitment of mothers who are participating in a randomized placebo-based clinical trial on the prevention of transmission of MS infections from mother to child in Bauru, Brazil, and (2) to present a rationale for conducting these trials by performing cross-cultural and temporal comparisons of documented levels of infection by the MS in mothers of Bauru, Brazil, with MS levels previously reported in mothers from industrialized countries.

## 2. Material & Methods

### 2.1. Sample Description of Mothers from Bauru, Brazil

A total of 1422 mothers were initially identified at a municipal maternal milk center in the city of Bauru, state of São Paulo, Brazil, between September of 2001 and July of 2003. During this period, mothers were visited at their domiciles and invited to participate in the research study in order to apply eligibility criteria. Those who agreed to be screened signed a consent form approved by institutional review boards at the University of Pittsburgh and the Universidade de São Paulo. A total of 693 first-time mothers were screened for MS when their babies were 2 months old. Participants were predominantly of a low socioeconomic status and were on average 20 years old (range of 16 to 35 years old).

### 2.2. Eligibility Criteria

First-time mothers who were the primary caretakers of the child were invited to participate. Only mothers who harbored at least 106  CFU of MS per ml of saliva, as determined by the Dentocult SM Strip Mutans test (see below), were included in the study. Eligibility criteria also excluded mothers who (1) had less than 20 teeth, (2) received fluoride therapy in the previous 3 months, (3) were taking, on a continual basis, medication that reduces salivary flow (antidepressants), or required antibiotic prophylaxis when examined by the dentist, and (4) presented with cognitive-medical compromise or whose child presented with similar conditions.

### 2.3. Mutans Streptococci Salivary Assay

This study employed the Dentocult SM Strip Mutans test (Orion Diagnostica, Espoo, Finland) to assess salivary levels of the MS in mothers. This assay is a simplified method that takes advantage of the MSB medium described by Gold and collaborators [16] which is traditionally employed to enumerate salivary levels of the MS. Mothers were asked to chew on paraffin for 1 minute and to discard the excess saliva. Subsequently, two thirds of an especially treated plastic strip were inserted into the mouth and rotated on the surface of the tongue about 10 times [17]. The strip was placed into a culture vial containing MSB medium and processed according to the manufacturer's instructions. Results were on a scale of 0 to 3 as follows: 0-1 <10^5^ CFU/mL of saliva, score 2 ≥10^5^-<10^6^ CFU/mL of saliva, and score of 3 ≥10^6^ CFU/mL of saliva.

### 2.4. Selection of Studies Reporting on Salivary Levels of the Mutans Streptococci in Mothers

Selection of studies for cross-cultural and temporal comparisons was primarily based on the use of MSB media by the traditional culturing method or by the Dentocult SM Strip Mutans test. This is so because this assay employs MSB medium and it has been shown to yield results highly comparable with the traditional culturing method that employs MSB medium [17]. Additional criteria included establishing cutoff levels of the MS at ≥10^5^ CFU/mL based on the ability to extract these cutoff levels from existing literature. If we were to use the cutoff point for the MS of ≥10^6^ CFU/mL of saliva, comparisons with previous studies would not have been possible with the exception of the Kohler study.

### 2.5. Statistical Analysis

Descriptive statistics and statistical analysis were performed with the use of SAS statistical software package (SAS, Cary, North Carolina, USA). An overall summary measure was computed by the Dersimonian-Laird test for proportions. A linear test for proportions was employed to test for trends across time. *Z*-tests were used to test for differences between two proportions.

## 3. Results

Table 1 shows the number of mothers whose domiciles were visited, those who were screened, and the number of eligible mothers. A total of 1422 domiciles were visited and 693 mothers were initially screened to apply eligibility criteria. Twenty-four % of mothers (168 of 693) were eligible to participate and were actually enrolled into the study. Review of the data revealed the most common issues for nonenrollment and refusal to participate in the study (Table 2). The most common reasons for nonenrollment were not having high salivary levels of the MS, that is, a score <3 (39.3%), noncontact with potential participants (29.2%), and mother not being the primary caretaker of the child (12.3%). The overall refusal rate for the study was 3.7%. 

Table 3 presents the prevalence rates for MS salivary levels ≥10^5^ CFU/mL for the present study and for studies conducted in the past in industrialized countries. A negative coefficient for the linear test indicated a significant decline (*P* < .0001) in the levels of the MS in mothers overtime; however the present study was not included in the analysis. Intercountry comparisons for mothers' salivary levels of the mutans streptococci using the study conducted in Bauru as the reference revealed significant differences with studies conducted in the late 1980s and 1990s. There were no significant differences between prevalence rates of the study conducted in Bauru and studies conducted in the late 1970s.

## 4. Discussion

This study is the first to report issues related to recruitment of women into studies aimed at the suppression of MS infections in mothers. The most common reason for not enrolling into this study was a mother not presenting with high salivary levels of the MS (Table 2). Yet, we were still able to enroll 168 mothers who met eligibility criteria because of the presence of levels of the MS compatible with the diagnosis of an infection by these organisms, that is, >10^6^ CFU/mL of saliva. Unfortunately, we were unable to assess the recruitment performance of completed trials aimed at suppressing salivary levels of the mutans streptococci in mothers.

The refusal rate to participate in this study was very low (3.7%), whereas studies cited in the medical literature report refusal rates ranging from 37% to 54% [18]. Given the fact that this is a population of low socioeconomic status, this refusal rate is surprising because, based on our experience, the decision-making process of mothers who recently delivered may affect willingness to enroll into a research study. In contrast, since saliva sampling and other dental-related protocols are likely to be perceived as less threatening than medical procedures, then the refusal rates observed in this study are more justifiable.

The importance of strategies for the suppression of MS in mothers for caries prevention in their children has been long recognized. In the only randomized controlled clinical trial to test this hypothesis, investigators were unable to demonstrate an effect of short-term treatment with an iodine-NaF solution on the transmission of the MS from mother to child [19]. But the entry level for the mutans streptococci was >2.5 × 10^4^ CFU/mL of saliva, and, as such, many of these mothers would not have been qualified for participation in the Swedish study conducted successfully by Kohler and colleagues three decades ago. Thus, the inability to duplicate the Swedish study could be due to the fact that the selected mothers were not highly infectious for MS.

These findings, coupled with observations on the declining levels of MS in the saliva of subjects from industrialized countries [20, 21], suggest that studies of the type performed by the Swedish group would be difficult to perform nowadays. They probably could not be performed again in Sweden as the MS levels for teenagers have declined significantly in recent years concomitant with the decline in caries scores [21]. If this decline, in both the caries rate and the MS levels, is reflective of the situation in industrialized countries, then interceptive studies are difficult to perform in order to replicate the Kohler studies, unless they were conducted in populations that have not realized the benefits of fluoridation and/or that are highly infected by the MS or unless reemergence of these infections in industrialized countries associated with ecological or environmental changes may lead to enhanced exposure and transmission of MS infections. This latter scenario is evident in studies conducted in Sweden where adolescent immigrants presented with higher caries rates than nonimmigrants [22]. 

Our cohort when compared with cohorts of studies aimed at determining the salivary levels of the MS showed that moderate-to-high levels of infection by the MS are still found nowadays at a high rate (Table 3) in the population of mothers of Bauru, Brazil**.** Moderate-to-high infection levels of the MS in Brazilian mothers are comparable to levels in mothers where initial interventions for curtailing the acquisition of MS by infants were conducted three decades ago. Based on the extensive documentation that mothers are the primary source of MS for the child [23–26], then expectant mothers of the city of Bauru would be a suitable population to perform studies to interfere with the transfer of MS from mothers to children. Lastly, the high rates of infection by the MS that are paralleled by high caries rates in this population make the implementation of clinical trials focusing on the suppression of salivary levels of the MS in mothers appealing. This is so because, if these trials are successful (or not), the results are readily interpretable and therefore applicable to segments of the population worldwide where oral health disparities are still a reality.

## Figures and Tables

**Table 1:** Number of mothers visited, screened, and eligible.

	*N*	%
Visited	1422	100%
Screened	693	49% (693/1422)
Eligible	168	24% (168/693)

**Table 2:** Issues of Nonenrollment and refusal to participate in the study.

Issue	*N*	%
Mutans assay score <3	493	39.3%
Mother not home at visit	367	29.2%
Mother not the primary caretaker	155	12.3%
Mother's address incorrect	93	7.4%
Lack of interest	46	3.7%
Mother wears full-arch prosthesis	32	2.6%
Mother's domicile not accessible	23	1.8%
Not First-time mother	10	<1%
Mother/child medical compromise	11	<1%
Mother traveling	8	<1%
Fluoride previous 3 months	7	<1%
Mother attending school	3	<1%
Not residing in the domicile	2	<1%
Newborn deceased	2	<1%
Mother leaving Bauru	1	<1%
Mother <20 teeth	1	<1%

Total	1254	

**Table 3:** Studies on the prevalence rates of salivary levels of the mutans streptococci in mothers.

Author/year	Country	Age of infants	Mothers screened (*N*)	MS ≥10^5^ CFU/mL Saliva—*N* (%)	*P*_STUDY_-*P*_BAURU_	Sig. level
Bauru 2001–06	Brazil	2 mos.	693	525 (76%)		
Kohler et al. 1978–81 [1]	Sweden	3–8 mos.	249	198 (80%)	4%	0.118
Brown et al. 1984-85 [5]	Australia	<2 yrs	112	91 (81%)	5%	0.118
Paunio et al. 1986–88 [6]	Finland	5–8 mos.	154	77 (50%)	−26%	<0.0001
Soderling et al. 1991 [8]	Finland	3 mos.	338	195 (58%)	−18%	<0.0001
Brambilla et al. 1993 [7]	Italy	not born	310	65 (21%)	−55%	<0.0001

[  ]: reference cited.
